# DynHeter-DTA: Dynamic Heterogeneous Graph Representation for Drug-Target Binding Affinity Prediction

**DOI:** 10.3390/ijms26031223

**Published:** 2025-01-30

**Authors:** Changli Li, Guangyue Li

**Affiliations:** School of Artificial Intelligence, Nanjing University of Information Science & Technology, Nanjing 210044, China; 202212620007@nuist.edu.cn

**Keywords:** drug-target binding prediction, heterogeneous graph, graph neural networks, graph representation learning

## Abstract

In drug development, drug-target affinity (DTA) prediction is a key indicator for assessing the drug’s efficacy and safety. Despite significant progress in deep learning-based affinity prediction approaches in recent years, there are still limitations in capturing the complex interactions between drugs and target receptors. To address this issue, a dynamic heterogeneous graph prediction model, DynHeter-DTA, is proposed in this paper, which fully leverages the complex relationships between drug–drug, protein–protein, and drug–protein interactions, allowing the model to adaptively learn the optimal graph structures. Specifically, (1) in the data processing layer, to better utilize the similarities and interactions between drugs and proteins, the model dynamically adjusts the connection strengths between drug–drug, protein–protein, and drug–protein pairs, constructing a variable heterogeneous graph structure, which significantly improves the model’s expressive power and generalization performance; (2) in the model design layer, considering that the quantity of protein nodes significantly exceeds that of drug nodes, an approach leveraging Graph Isomorphism Networks (GIN) and Self-Attention Graph Pooling (SAGPooling) is proposed to enhance prediction efficiency and accuracy. Comprehensive experiments on the Davis, KIBA, and Human public datasets demonstrate that DynHeter-DTA exceeds the performance of previous models in drug-target interaction forecasting, providing an innovative solution for drug-target affinity prediction.

## 1. Introduction

Drug-target affinity prediction (DTA) plays an important role in drug efficacy prediction, toxicity assessment, and drug metabolism research [1]. Experimentally, affinity is typically measured using dissociation constants, inhibition constants, or half-maximal inhibitory concentration. However, traditional methods for obtaining DTA in the laboratory are often time-consuming and resource-intensive. Researchers may spend decades and significant resources classifying, screening, and experimenting with drugs [2,3,4]. Therefore, the development of accurate computational methods for predicting DTA would provide valuable insights for drug discovery, guiding the optimization and improvement of drug molecules, enhancing their selectivity, potency, and safety, and thus significantly accelerating the drug discovery process.

Methods for predicting DTA are categorized into the following three major groups: structure-focused methods, machine learning approaches, and deep learning-driven techniques [5]. Conventional structure-driven DTA prediction approaches mainly involve molecular docking [6] and molecular dynamics simulations [7], both of which rely on high-precision 3D structural data of the drug and protein to model their interactions. Although these methods have yielded promising results in terms of predictive performance, they require highly accurate structural information of both molecules and proteins, and consume substantial computational resources, which limits their large-scale application.

To tackle these problems, machine learning (ML)-based techniques have gained prominence [8,9,10]. These approaches do not require high-precision structural information of the drug and protein, but instead use textual information from the drug and protein sequences for DTA prediction. Specifically, they extract primary features from the drug and protein sequences and input these features into classifiers, such as decision trees [11] or support vector machines [12], for training, to obtain drug–protein interaction models, which are then used for prediction. Some classical methods include kernel-based methods like KronRLS [13] and similarity-based methods like SimBoost [14]. However, these early machine learning approaches often require significant domain expertise to construct complex feature engineering.

Currently, deep learning-based prediction methods have demonstrated significant advantages in DTA prediction [15,16,17]. Predicting DTA requires consideration of the complex molecular structures and interactions of both the drug and protein. Deep learning can process large volumes of high-dimensional biological data, efficiently managing this complexity by automatically learning and extracting features, without the need for manual feature extraction or precise predictions. This approach reduces information loss and improves the robustness and generalization ability of prediction models. For example, DeepDTA [18] uses convolutional neural networks (CNNs) to separately extract representations of drug and protein, then concatenates these representations and passes them through FC layers to obtain predictions of the drug–protein binding affinity. WideDTA [19] extends DeepDTA by adding two additional CNNs to model the interaction strength between individual protein substructures and drug substructure pairs. TransformerCPI [20] utilizes transformers [21] to capture sequence data from both drugs and proteins. Although deep learning models have demonstrated strong effectiveness in drug-target affinity prediction, several issues remain, such as the following: On the one hand, these sequence-based convolutional methods require equal lengths for input sequences, which necessitates truncating drug and protein sequences to a unified length, potentially leading to loss of information from both the drug and protein. On the other hand, atoms in distant regions of a drug molecule or amino acid residues in close proximity within a protein sequence may be spatially close in reality, but the spatial structural features of drug compounds and protein structures cannot be captured by these deep learning models.

To address these challenges, more researchers are adopting graph neural network (GNN)-based approaches for drug-target affinity prediction [22,23,24]. GNNs offer unique advantages in DTA prediction, as both drugs and receptor proteins can be treated as complex graph structures, with interactions between the drug and protein modeled as interactions within a complex graph. GNNs are able to effectively capture the intricate molecular structures of drug molecules and protein molecules, as well as the interactions between them. For example, GraphDTA [25] constructs a drug molecular graph, with atoms representing the nodes and the bonds between atoms serving as the edges, thus enriching the representation of drug molecules. DGraphDTA [26] further builds a protein graph using contact map methods. It treats amino acid residues as nodes and considers them to be connected if the distance between them is below a certain threshold.

Although the aforementioned DTA prediction methods have achieved good results, they primarily focus on the features of individual drug and protein molecules while neglecting the relationships between drugs and proteins. For instance, HGRL-DTA [27] integrates drug–protein interactions into the features of drugs and proteins but overlooks drug–drug and protein–protein relationships. MSF-DTA [28] only considers protein–protein relationships, ignoring the relationships between drug–drug and drug–protein. To address these issues, this paper proposes an end-to-end DTA prediction method, DynHeter-DTA, that makes several contributions, which are as follows:

- First, a mechanism was designed to dynamically adjust the connection strengths between drug–drug, protein–protein, and drug–protein interactions, constructing a variable heterogeneous graph structure in order to deeply explore drug-target interactions.
- Furthermore, a model leveraging Graph Isomorphism Networks (GIN) and Self-Attention Graph Pooling (SAGPooling) techniques for drug-target interaction forecasting was introduced, improving both prediction efficiency and accuracy.
- Finally, comprehensive experiments were conducted on the Davis, KIBA, and Human public datasets, comparing multiple baseline models. The proposed model’s superior performance was validated, providing a new approach for drug affinity prediction.

## 2. Results and Discussion

### 2.1. Settings

In this study, the dataset was divided into training and test sets with a ratio of 5:1, and fivefold cross-validation was applied to the training set. The proposed model DynHeter-DTA and comparison methods were trained using the training set and validated on the test set. Experiments were carried out on a machine equipped with 16 GB of VRAM on an NVIDIA MSI 4080 GPU and an Intel 11400f CPU, all from Nanjing, Jiangsu, China. The model was implemented with PyTorch 2.4.1, torch-geometric 2.6.1, and RDKit 2024.3.5, trained with the Adam optimizer at a fixed learning rate of 0.0005 and a batch size of 512. All hyperparameters, including the number of training epochs used in our experiments, are specified in Table 1.

### 2.2. Metrics

Mean squared error (MSE) is used to quantify the discrepancy between the model’s predicted values and the ground truth, thereby evaluating DynHeter-DTA’s prediction performance. The formula is as follows:

(1) $$MSE=\frac{1}{n}\sum_{i=1}^{n}{\left(y_{i}-{\hat{y}}_{i}\right)}^{2}$$

where n is the size of the test set, and $y_{i}$ and ${\hat{y}}_{i}$ are the actual and predicted values of the test sample, respectively.

The Concordance Index (*CI*) is applied to evaluate the model’s accuracy and consistency in predicting the ranking of outcomes. The formula is as follows:

(2) $$CI=\frac{1}{Z}\underset{d_{x}-d_{y}}{\sum }h\left(b_{x}-b_{y}\right)$$

(3) $$h\left(x\right)=\left\{\begin{array}{c} 1,if\;x>0\\ 0.5,if\;x=0\\ 0,if\;x<0 \end{array}\right.$$

where Z is a normalization constant and $h\left(x\right)$ is the step function.

The Relative Coefficient of Determination ($r_{m}^{2}$) is an indicator used to assess the relative goodness-of-fit of a regression model. It is a modified version of the coefficient of determination, considering the number of independent variables used in the model. The formula for the Relative Coefficient of Determination is as follows:

(4) $$r_{m}^{2}=r^{2}\times \left(1-\sqrt{r^{2}-r_{0}^{2}}\right)$$

where $r^{2}$ and $r_{0}^{2}$ are the squared correlation coefficients with and without an intercept, respectively.

These above three evaluation metrics enable us to thoroughly assess the model’s performance and its predictive power in practical applications.

For the binary classification task of drug-target interaction (DTI), the following evaluation metrics are used in this study:

(5) $$Precision=\frac{TP}{TP+FP}$$

(6) $$Recall=\frac{TP}{TP+FN}$$

Here, TP, FP, and FN represent the number of true positives, false positives, and false negatives, respectively. In addition to accuracy and recall, the proposed model is also evaluated using the area under the receiver operating characteristic curve (AUROC) and the area under the precision-recall curve (AUPR).

### 2.3. Model Performance

To evaluate DynHeter-DTA’s performance, it was compared with baseline models, including KronRLS [13], SimBoost [14], DeepDTA [18], WideDTA [19], GraphDTA [25], MGraphDTA [29], GEFA [30], WGNN-DTA [31], and DGraphDTA [26]. A detailed comparison and analysis of each model’s performance on different datasets was conducted. The performance results and a summary of the comparison with the baseline methods are presented in Table 2 and Table 3.

As demonstrated in Table 3, DynHeter-DTA exceeds the performance of other methods on the Davis dataset, achieving values of 0.130, 0.923, and 0.828 in MSE, CI, and $r_{m}^{2}$, respectively. Compared to the second-best model, DGraphDTA, our model improves these metrics by 7.2%, 1.9%, and 12.8%, respectively. As shown in Table 4, DynHeter-DTA also has a strong performance on the KIBA dataset, achieving values of 0.123, 0.908, and 0.821 in MSE, CI, and $r_{m}^{2}$, respectively. In comparison to the second-best model, DGraphDTA, DynHeter-DTA improves these metrics by 0.3%, 0.4%, and 4.0%, respectively.

For the DTI task, as is shown Table 4, the proposed model is compared to GCN [32], GanDTI [33], GraphCPI [34], MGraphDTA [29], and TransformerCPI [20]. DynHeter-DTA achieves AUC, Precision, Recall, and F1 scores of 0.988, 0.956, 0.961, and 0.951, respectively, which show improvements of 0.5%, 2.2%, 1.4%, and 0.8% over the best-performing model, MGraphDTA. These results demonstrate that DynHeter-DTA has a high performance and potential within the domain of drug–protein affinity prediction.

### 2.4. Binding Affinity Prediction on Testing Data

**Figure 1:**
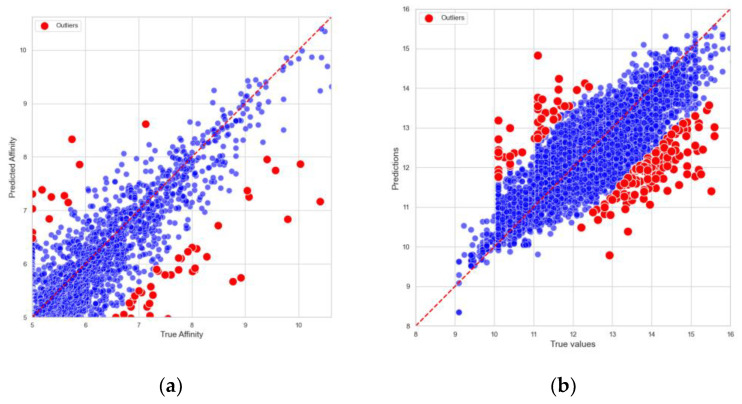
Comparison between predicted and actual affinity values. (**a**) Comparison of true and predicted values on Davis dataset; (**b**) comparison of true and predicted values on KIBA dataset. Outliers are depicted in red, and normal values are depicted in blue.

To further assess the reliability of the predictions, predicted binding affinities were compared to the actual binding affinities from the Davis and KIBA datasets on the test set. As shown in Figure 1, DynHeter-DTA demonstrates an impressive performance. The predicted values exhibit a strong correlation with the true values, with the data points in the scatter plot closely distributed around the ideal prediction line, indicating that the model fits the actual data very well. This further validates the accuracy and stability of the model.

In order to systematically investigate the origins of outliers, this study analyzed the data points in the Davis and KIBA datasets whose prediction errors ranked in the top 5%. The results show that the average absolute errors for the entire datasets were 0.178 and 0.159, respectively, whereas these outliers (accounting for about 5.01% of the samples) exhibited average absolute errors of 1.233 and 0.1124, which are significantly higher than the overall levels. This finding indicates that utilizing the 95th percentile threshold effectively captures the high-error tail, which substantially deviates from the mean.

Within the context of drug–protein binding prediction, grouping outliers by compounds and proteins revealed that most anomalies originated on the compound side rather than the protein side. Notably, compounds 11,409,972, 11,984,591, 16,038,120, 126,565, and 11,409,972 displayed average errors exceeding 1 across multiple targets. For instance, compound 16,038,120 not only showed a large deviation with AK (2.14), but also with BMPR1B (1.82), TSSK1B (1.80), TRKB (1.57), and CHEK1 (1.49). Similarly, compound 11,409,972 showed high errors for EPHB6 (2.31), MET (1.90), LOK (1.66), and TNK1 (1.65). These findings suggest that such compounds do not merely “fail” on a single target, but rather exhibit consistently high errors across multiple targets.

To further elucidate the underlying causes, an in-depth analysis of the structures and physicochemical properties of these compounds was conducted. The results indicate that their scaffolds and substructures differ markedly (low Tanimoto similarity), implying that they are not derivatives of a single series that might cause systematic bias. However, at the macroscopic level of physicochemical features, these compounds commonly exhibit high molecular weights (>500 Da), elevated LogP values (>4), and multiple aromatic rings and hydrogen-bonding sites, collectively manifesting as “complex, lipophilic, and high-molecular-weight” attributes.

### 2.5. Ablation Study

To examine the key determinants of the DynHeter-DTA’s predictive capability, ablation experiments were executed using different variants of the model. The subsequent numbered lists illustrate

1. Without heterogeneous graph, using GCN instead of GIN and SAGPooling;
2. Without heterogeneous graph, using GIN and SAGPooling;
3. Using dynamic heterogeneous graph with GIN and SAGPooling.

Through these ablation experiments, it was found that the key factors affecting the model’s predictive performance are the use of heterogeneous graphs and the application of GIN and SAGPooling. On the Davis and KIBA datasets, the model performed the worst when the heterogeneous graph was not used and GCN replaced GIN and SAGPooling. When GIN and SAGPooling were used without the heterogeneous graph, the model performance improved. However, the best performance across all metrics was achieved when the dynamic heterogeneous graph was used in conjunction with GIN and SAGPooling. This result demonstrates that the introduction of the dynamic heterogeneous graph and the synergistic effect of GIN and SAGPooling significantly enhanced the model’s predictive capabilities, highlighting the critical role these components play in improving model performance. The experimental results are shown in Table 5 and Table 6.

### 2.6. Visual Explanation

#### 2.6.1. Visualization of the Dynamic Heterogeneous Graph

To better understand the proposed variable heterogeneous graph prediction model, a visual analysis of the constructed drug–protein heterogeneous graph is conducted. In traditional static graph structures, the edges are usually based on predefined similarity or interaction strength. However, in our model, the edges are adaptively optimized according to the features of the nodes. This dynamic adjustment mechanism allows the graph structure to continuously evolve during training, providing a more accurate reflection of the affinity relationship between drugs and proteins.

**Figure 2:**
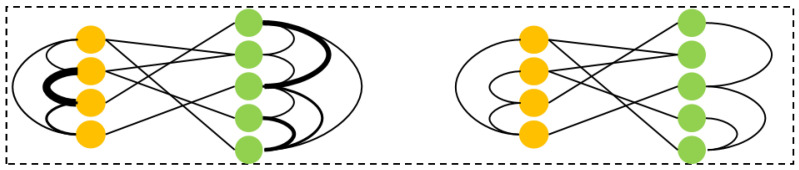
Example of graph structure before and after dynamic adjustment. In this figure, the thickness of the edges indicates the strength of edge weights. Thicker edges represent higher weights, while thinner edges indicate lower weights.

As shown in Figure 2, the nodes in the graph are divided into the following two categories: drug nodes and protein nodes. The edges between drug nodes represent drug-to-drug similarity, the edges between protein nodes represent protein-to-protein similarity, and the edges between drug and protein nodes represent their interaction relationship. This heterogeneous graph structure enables us to capture the multi-level relationships between drugs and proteins comprehensively.

**Figure 3:**
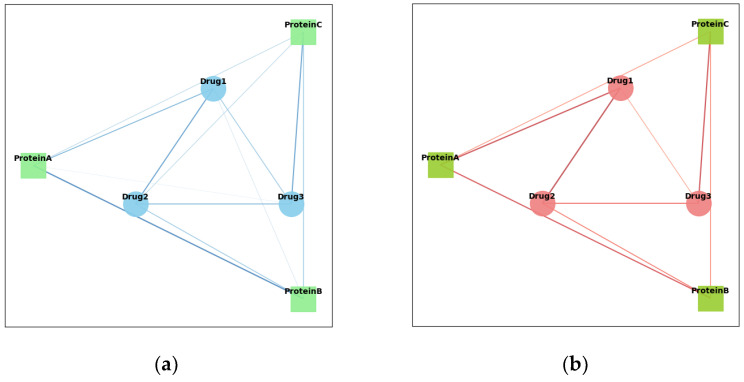
Comparison between the initial heterogeneous graph and the processed heterogeneous graph. (**a**) Initial heterogeneous graph; (**b**) the processed heterogeneous graph.

To further illustrate the dynamic changes in the graph, several drug molecules and protein molecules were selected to form a heterogeneous graph for dynamic adjustment. The comparison between the initial graph structure and the dynamically adjusted graph structure is displayed in Figure 3. The initial graph provides the basic interactions between drugs and proteins, while the dynamically adjusted graph highlights the interactions that the model considers more important by optimizing the edge weights. This optimization not only enhances the expressive power of the graph, but also improves the model’s ability to adapt to complex data, providing more effective information support for drug–protein affinity prediction.

#### 2.6.2. Visualization of GIN and SAGPooling

**Figure 4:**
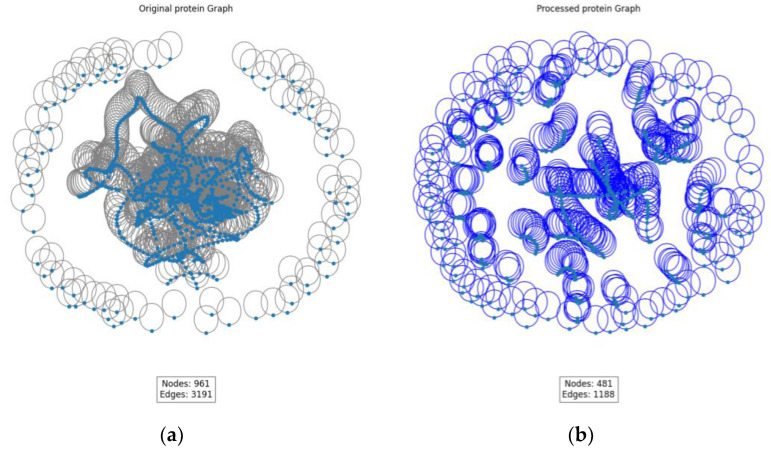
Processing of the first protein graph in Davis dataset. The blue nodes represent amino acid residues, and the circles represent the connections between these amino acid residues. (**a**) Initial protein graph; (**b**) protein graph after GIN and SAGPooing.

As shown in Figure 4, a visual analysis of GIN and SAGPooling was conducted. The left side presents the original graph, while the right side displays the processed graph. By comparing the two graphs, significant changes in the graph structure and feature representation after applying GIN and SAGPooling are clearly observable.

GIN significantly enhances the node feature representations through its unique feature aggregation mechanism. Specifically, GIN aggregates the features of neighboring nodes by weighted summation at each layer and applies a multi-layer perceptron (MLP) for non-linear transformation. This ensures that the feature representation of each node contains not only its own information, but also the features of its neighboring nodes. This method effectively captures subtle relationships in the graph, ensuring that information is fully propagated across the graph structure.

In the original graph, the number of nodes is denoted as 961 and the number of edges as 3191. After the pooling operation, the number of nodes is reduced to 481 and the number of edges to 1188. This reduction is a result of the SAGPooling operation, which selects and retains the most important nodes based on an attention mechanism. GIN enhances the feature representations of the nodes during feature extraction, making it more effective in identifying and retaining critical nodes during the pooling process. This combination of GIN and SAGPooling significantly improves the node feature representations in the model while reducing irrelevant information and redundant nodes and edges. As a result, the model can more efficiently learn the affinity relationships between drugs and proteins.

#### 2.6.3. Visualization of Drug–Protein Binding

**Figure 5:**
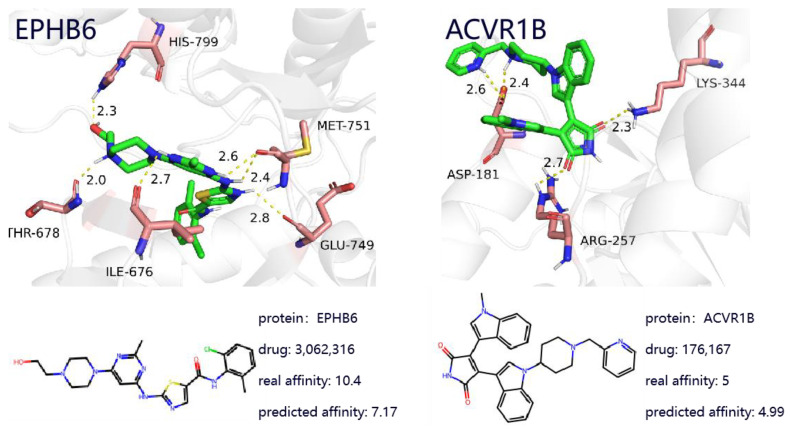
The binding affinity values between the drugs and the targets.

To further investigate the effectiveness of the proposed method, this study presents two case studies of drug-target complexes, which provide deeper insight into DTA prediction and contribute to understanding the potential mechanisms behind protein-target-based drug discovery. As shown in Figure 5, the left panel illustrates the complex between the targeted drug Dasatinib and the gene EPHB6, while the right panel shows the complex between the targeted drug Enzastaurin and the target ACVR1B. By utilizing known drug-target binding examples, the effectiveness and practicality of the proposed model in real-world applications are validated.

### 2.7. Discussion

In this study, we propose a drug-target affinity prediction model trained on the DAVIS and KIBA datasets, demonstrating broad potential for real-world applications. While the model exhibits a limited performance on certain complex, lipophilic compounds of high molecular weight that contain multiple aromatic rings and hydrogen-bonding sites, it effectively predicts the binding affinities between novel drug molecules and their target proteins in most other cases. This capability assists in identifying lead compounds during drug discovery and offers valuable insights for drug development. Furthermore, it can uncover previously unknown protein–drug interactions and supports the identification of new therapeutic targets.

Although the model may require further optimization for more complex tasks, such as multi-target drug design, its methodological framework exhibits strong scalability. In the future, it could be applied to other areas, such as protein–protein interaction prediction, drug–drug interaction analysis, and protein–antibody interaction prediction, showing considerable potential for expansion in these fields.

## 3. Materials and Methods

**Figure 6:**
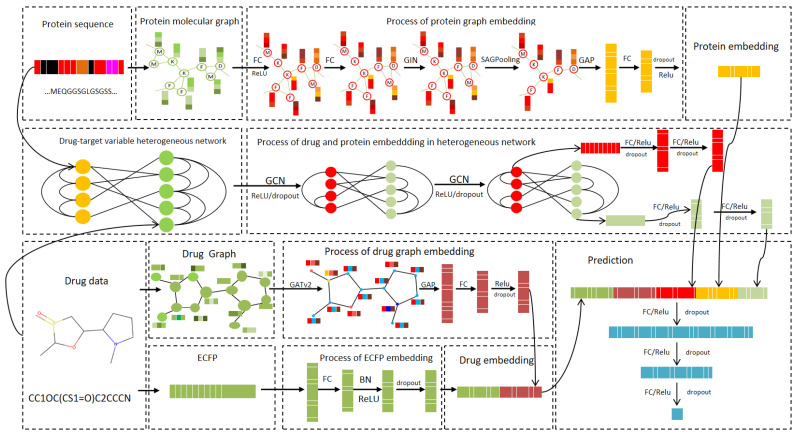
Architecture of the proposed DynHeter-DTA.

In this section, a dynamic heterogeneous graph representation learning model, DynHeter-DTA, for DTA prediction is proposed. The model’s input includes the drug and protein sequences, and the overall architecture is divided into the following two parts: feature extraction and relationship modeling.The entire model framework is illustrated in Figure 6.

In the feature extraction part, for drug embedding, the drug’s Morgan fingerprints (ECFP) [35] are first computed using the RDKit library, and the fingerprint embedding is extracted through an FC layer. The drug’s SMILES sequence is then converted into a molecular graph, and its features are extracted using a Graph Attention Network (GAT). The results are passed through an FC layer to generate the molecular graph embedding. These two embeddings are then concatenated to form the drug embedding. For protein sequences, the method proposed by Jiang et al. [26] is adopted, which transforms the protein sequence into a protein residue graph. GINs are employed to extract features, and a SAGPooling layer is applied to remove irrelevant nodes. The protein embedding is generated through global pooling.

In the relationship modeling part, a dynamic heterogeneous graph is constructed based on drug and protein data, integrating drug–drug and protein–protein similarities, and the complex interactions between drugs and proteins. A two-layer GCN is employed to extract global features for both the drug and protein. These global features are then fused with the previously extracted drug and protein embeddings and processed through FC layers. The final output is the predicted binding affinity score. 

### 3.1. Database

The Davis [36] and KIBA [37] datasets are two important resources for studying drug–protein interactions and are essential for drug discovery and progress in the biomedical field. The Davis dataset is based on experimentally determined drug–protein interaction data, containing detailed records of drug molecules, their associated protein targets, and their binding affinities, typically represented by Kd values. This dataset provides essential information about drug–protein interactions. On the other hand, the KIBA dataset focuses on the interactions between kinase inhibitors and protein kinases. It includes data on kinase inhibitor molecules and their binding affinities with kinase proteins, assisting in the prediction of potential kinase inhibitors’ effectiveness. The Davis and KIBA datasets are employed to evaluate the model in comparison to other baseline models. In addition, this paper also introduces the Human [38] dataset to evaluate the proposed model, which includes 3369 positive samples and 3359 negative samples. Table 7 summarizes the specific details of these three datasets.

In the Davis, KIBA, and Human datasets, drug molecules are represented by a structured SMILES notation. SMILES encodes the structure of a drug molecule as a string, where atoms and bonds are represented by specific characters. RDKit is a machine learning software library that assists in solving challenges related to chemical information; PyTorch is a free, open-source platform for deep learning applications and machine learning tasks. PyG is a graph neural network library built on top of PyTorch. The RDKit [39] library is employed to construct node and edge features for drug molecules, while the drug graph structure is built using PyG [40]. The model is developed using PyTorch [41].

In all datasets, proteins are represented by their sequences. The one-dimensional representation of protein sequences provides basic information about proteins, but it often neglects the critical role of spatial structure in protein functionality [42]. On the other hand, the three-dimensional structural representation of proteins is highly complex and is typically represented as a point cloud to capture the protein’s spatial conformation. While this representation can capture spatial information, it incurs significant computational cost [43]. To balance the consideration of spatial structure with computational efficiency, Jiang et al. [26] proposed a method to construct protein graph representations by predicting contact maps. In this representation, a protein is viewed as a graph, where nodes represent amino acids and edges indicate interactions between amino acids.

### 3.2. Drug–Protein Dynamic Heterogeneous Graph

The heterogeneous graph is an effective representation of the relationship between drugs and proteins. The heterogeneous graph is denoted as $G=\left\{H,A\right\}$, where H is the feature matrix and A is the adjacency matrix. The entire heterogeneous graph consists of the feature matrix as well as the edge weight matrix. The initial adjacency matrix is

(7) $$\hat{A}=\left[\begin{array}{cc} S_{d} & B\\ B^{T} & S_{p} \end{array}\right]$$

where $S_{d}\in R^{d_{r}\times d_{r}}$ is the drug–drug similarity matrix; $B\in R^{d_{r}\times d_{p}}$ is the drug–protein binding affinity matrix, and $B^{T}$ is its transpose; and $S_{p}\in R^{d_{p}\times d_{p}}$ is the protein–protein similarity matrix. $\hat{A}=A+I$ represents an adjacency matrix where each node has a self-loop. The matrices $S_{d}$ and $S_{p}$ are obtained using the following Tanimoto coefficients and global alignment methods, while B is provided by the dataset.

Here, the Tanimoto coefficient is used to measure drug–drug similarity [44]. Each drug is represented by its Morgan fingerprints to capture the local environmental information of its molecular structure, effectively describing the structural features of the molecule. Subsequently, the Tanimoto coefficient [43] is used to measure the resemblance between two drugs. The formula can be expressed as follows [44]:

(8) $$S_{d}\left(a,b\right)=\frac{a*b}{{\left\|a\right\|}^{2}+{\left\|b\right\|}^{2}-a*b}$$

where a and b represent the fingerprint vectors of two drug molecules. Furthermore, the value of the Tanimoto coefficient varies between 0 and 1; a value approaching 1 suggests greater structural similarity of the two molecules, and vice versa.

The Needleman–Wunsch (NW) global alignment algorithm [45] is used in this study to calculate protein–protein similarity. The NW algorithm is a classical dynamic programming approach designed to globally align two sequences and determine their optimal matching. It constructs a matrix to incrementally compute the similarity scores between the two sequences, and ultimately identifies the best alignment path through a backtracking procedure. During matrix filling, the algorithm considers match, mismatch, and gap insertions, ultimately producing a similarity score that reflects the global alignment between the sequences. The calculated similarity is denoted as $S_{p}$ in this study.

To capture the features of drug–protein interaction affinity in the heterogeneous graph, Graph Convolutional Networks (GCN) are utilized. This method uses the feature matrix and adjacency matrix of nodes as inputs. By performing convolution operations on the graph, it captures the feature relationships between nodes and their neighbors. The general learning process of GCN can be expressed as follows [32]:

(9) $$H^{\left(l+1\right)}=f\left(H^{\left(l\right)},A\right)=\sigma \left({\hat{D}}^{-\frac{1}{2}}\hat{A}{\hat{D}}^{-\frac{1}{2}}H^{\left(l\right)}W^{\left(l\right)}\right)$$

where $\hat{A}=A+I$ is the adjacency matrix with added self-loops, I is the identity matrix, $\hat{D}$ is the degree matrix of *A*, *σ* is the non-linear activation function (ReLU used in our study), $W^{\left(l\right)}$ is the trainable weight matrix of the l-th layer, and $H^{\left(l\right)}$ is the node feature matrix at the l-th layer, which is derived from the one-hot encoding of the drug and protein, along with their similarity and binding relationships.

By incorporating drug–drug similarity and protein–protein similarity into the heterogeneous graph, and designing a dynamic edge weight adjustment method, a variable heterogeneous graph for the drug–protein similarity affinity is constructed. The update of the heterogeneous graph is integrated with the model training. The model can more comprehensively capture the similarity and interaction information between drugs and proteins. This enhanced heterogeneous graph can dynamically learn the optimal graph structure, improving both the graph’s expressiveness and the model’s flexibility and adaptability. This, in turn, provides more effective informational support for drug–protein affinity prediction.

To further achieve this, two transformations to the heterogeneous graph adjacency matrix and node feature matrix are exerted, respectively, which result in the following dynamic heterogeneous graph learning process:

(10) $$H^{\left(l+1\right)}=f\left(H^{\left(l\right)},A\right)=\sigma \left({\hat{D}}^{-\frac{1}{2}}\varphi \left(\hat{A}\right){\hat{D}}^{-\frac{1}{2}}\psi \left(H^{\left(l\right)}\right)W^{\left(l\right)}\right)$$

The adjacency matrix of the designed variable heterogeneous graph with self-loops is

(11) $$\varphi \left(\hat{A}\right)=\left[\begin{array}{cc} ReLU\left(S_{d}-w_{1}\right) & B\\ B^{T} & ReLU\left(S_{p}-w_{2}\right) \end{array}\right]$$

where $w_{1}$ and $w_{2}$ directly affect the edge structure retained in the graph and are updated through back propagation.

The feature matrix of the designed variable heterogeneous graph is

(12) $$\psi \left(H\right)=\left[D,\varphi \left(\hat{A_{1}}\right)\right]$$

where $\varphi \left(\hat{A_{1}}\right)$ is obtained by marking all non-zero elements in matrix $\varphi \left(\hat{A}\right)$ as 1. This is

(13) $$\varphi \left(\hat{A_{1}}\right)=\left\{\begin{array}{c} 0,\;A\left(i,j\right)=0\\ 1,\;A\left(i,j\right)\neq 0 \end{array}\right.$$

where $D\in R^{\left(d_{r}+d_{p}\right)\times 2}$ is the one-hot encoding of drugs and proteins.

### 3.3. Drug Graph and Protein Graph Embedding Representation

A Graph Attention Network (GAT) is leveraged to learn the embedding from the drug graph [46]. For a node i in the drug graph $G_{d}=\left\{V_{d},A_{d}\right\}$ and its neighboring nodes j, the attention coefficient is

(14) $$e_{ij}=a\left(Wv_{i},Wv_{j}\right)$$

where $v_{i}$ and $v_{j}$ are the features of nodes i and j; a is the trainable attention weight vector; W is the trainable weight matrix used for linear transformation; and $e_{ij}$ represents the importance of node j to node i.

To facilitate a comparison between different adjacent nodes, the Softmax function is used over the neighbors.

(15) $$a_{ij}=\frac{exp\left(e_{ij}\right)}{\sum_{k\in N_{i}}exp\left(e_{ik}\right)}$$

where $N_{i}$ is the neighborhood of node i in the graph.

Then, by calculating the weighted sum of the features based on the normalized attention coefficients of each node, the output features are derived, as follows:

(16) $$v_{i}^{new}=\sigma \left(\underset{j\in N_{i}}{\sum }\alpha_{ij}Wv_{j}\right)$$

where σ is the non-linear activation function (ReLU in the model) and $G_{gat}$ represents the processed feature of the drug graph after applying GAT.

A Graph Isomorphism Network (GIN) is used to extract the embedding of the protein graph [47]. For a node in the protein graph and its neighboring nodes, the operation on the graph can be expressed by the following formula:

(17) $$v_{p}^{h+1}=MLP\left(\left(1+\epsilon \right)\cdot v_{p}^{h}+\underset{j\in N_{p}}{\sum }v_{q}^{h}\right)$$

where $N_{p}$ denotes the set of neighboring nodes of node p in the protein graph, and $v_{p}^{h}$ and $v_{q}^{h}$ are the feature vectors of nodes p and q in the protein graph at the *h*-th layer, respectively. Additionally, ϵ is a learnable parameter and $G_{pgin}$ refers to the protein graph after processing with GIN.

SAGPooling [48] is a node feature aggregation technique based on the self-attention approach, capable of adaptively learning the importance weights of each node, and thus effectively distinguishing which nodes should be retained and which can be discarded. The process is as follows. First, calculate the importance score

(18) $$s=GNN\left(X_{pgin},A_{pgin}\right)$$

where $X_{pgin}$ and $A_{pgin}$ represent the feature matrix and adjacency matrix of the protein graph after processing with GIN, respectively. Then, select the top *K* highest-scoring nodes

(19) $$i=top_{k}\left(s\right)$$

Finally, the node features and the adjacency matrix are updated according to the following rules:

(20) $$X_{pgin}^{new}=X_{pgin}[i]$$

(21) $$A_{pgin}^{new}=A_{pgin}[i,i]$$

### 3.4. Information Fusion

The node features in the heterogeneous graph are subsequently fused with the information from the drug graph and the protein graph, respectively. Specifically, given the feature $h_{v}$ of the drug node v in the heterogeneous graph, the processed molecular graph $G_{gat}$, and the processed drug ECPR representation $e_{v}$, the enhanced embedding of the drug node can be defined as follows:

(22) $$h^{d}=e_{v}∥\left[GAP\left(G_{gat}\right)\right]∥\left[MLP\left(MLP\left(h_{v}\right)\right)\right]$$

where ∥ represents the concatenation process and GAP refers to global average pooling.

Similarly, given a protein node u in the heterogeneous graph, with the feature $h_{u}$ and the processed protein graph $G_{sag}$, the fused representation of the protein node can be defined as

(23) $$h^{t}=\left[GAP\left(G_{sag}\right)\right]∥\left[MLP\left(MLP\left(h_{u}\right)\right)\right]$$

### 3.5. DTA Prediction

In the model, predicting the drug–protein binding affinity is treated as a regression problem. By concatenating the representation $h^{d}$ of drug d and the representation $h^{t}$ of protein t, the final prediction is made through three fully connected layers. This approach effectively integrates the features of both drug and protein, enhancing the model’s prediction accuracy. The formula is as follows:

(24) $$\hat{y_{dt}}=MLP\left(MLP\left(MLP\left(h^{d}∥h^{t}\right)\right)\right)$$

The regression loss function, mean squared error (MSE), quantifies the difference between the predicted values and the ground truth. The formula is as follows:

(25) $$MSE=\frac{1}{n}\sum_{i=1}^{n}{\left(y_{dt}-\hat{y_{dt}}\right)}^{2}$$

where $y_{dt}$ is the true binding affinity.

## 4. Conclusions

For drug–protein binding prediction, this paper addresses two main issues. The first issue is how to effectively utilize the connections between drugs and proteins. To tackle this, we propose a dynamic heterogeneous graph structure. It can adaptively learn the optimal graph structure and dynamically adjust the connection strengths between drug–drug, protein–protein, and drug–protein interactions, thereby more effectively capturing complex interaction relationships.

The second issue is that in reality, the number of drugs is much smaller than the number of proteins, and in the protein–drug binding process, there are likely many irrelevant or weakly influential nodes on the protein. To address this, we combine GIN and SAGPooling techniques to accurately extract the important node features from proteins, simplify the protein graph structure, and enhance both prediction efficiency and accuracy.

Experiments on the Davis, KIBA and Human datasets were conducted to evaluate the performance of DynHeter-DTA. The model demonstrated substantial improvements over existing methods in both prediction accuracy and its ability to capture complex relationships. The comparison between predicted and true binding affinities showed a high correlation, validating the effectiveness of our approach. An ablation study was conducted to evaluate the contribution of each component of the model, revealing that both the dynamic graph structure and the feature extraction techniques (GIN and SAGPooling) are crucial for enhancing performance. Moreover, a visual explanation of the model’s decision-making process further confirmed that DynHeter-DTA successfully identifies important drug-target interaction features, offering insights into the underlying binding mechanisms. These results emphasize the potential of DynHeter-DTA as a powerful tool for drug affinity prediction.

## Figures and Tables

**Table 1:** Hyperparameters used in our experiments.

Hyperparameters	Setting
Epoch	4000
Batch size	512
Optimizer	Adam
Learning rate	0.0005
Dropout rate of heterogeneous graph, drug and protein graph, ECFP	0.2/0.3/0.5
Embedding size of heterogeneous graph, drug and protein graph, ECFP	256/1024/128
Fully connected layers	1024/512/1
$w_{1}$ and $w_{2}$	0.1/0.1

**Table 2:** The model performance results on Davis dataset.

Model	MSE	CI	$r_{m}^{2}$
KronRLS	0.379	0.871	0.407
Simboost	0.282	0.872	0.664
DeepDTA	0.261	0.878	0.630
WideDTA	0.262	0.886	-
GraphDTA	0.229	0.893	-
GEFA	0.228	0.893	-
MGraphDTA	0.207	0.900	0.710
WGNN-DTA	0.208	0.900	0.692
DGraphDTA	0.202	0.904	0.700
DynHeter-DTA	0.130	0.923	0.828

**Table 3:** The model performance results on KIBA dataset.

Model	MSE	CI	$r_{m}^{2}$
KronRLS	0.441	0.782	0.342
Simboost	0.222	0.836	0.629
DeepDTA	0.194	0.863	0.673
WideDTA	0.179	0.875	-
GraphDTA	0.139	0.891	-
MGraphDTA	0.128	0.902	0.801
WGNN-DTA	0.144	0.885	0.781
DGraphDTA	0.126	0.904	0.786
DynHeter-DTA	0.123	0.908	0.821

**Table 4:** The model performance results on Human dataset.

Model	AUC	Precision	Recall	F1 Score
GCN	0.956	0.862	0.928	-
GanDTI	0.982	0.943	0.941	0.942
GraphCPI	0.973	0.940	0.890	-
MGraphDTA	0.983	0.934	0.947	0.943
TransformerCPI	0.974	0.928	0.939	0.933
DynHeter-DTA	0.988	0.956	0.961	0.951

**Table 5:** Ablation experiments on Davis dataset.

Model	MSE	CI	$r_{m}^{2}$
Without heterogeneous graph, using GCN instead of GIN and SAGPooling	0.257	0.885	0.660
Without heterogeneous graph, using GIN and SAGPooling	0.229	0.892	0.695
Using dynamic heterogeneous graph with GIN and SAGPooling	0.130	0.923	0.828

**Table 6:** Ablation experiments on KIBA dataset.

Model	MSE	CI	$r_{m}^{2}$
Without heterogeneous graph, using GCN instead of GIN and SAGPooling	0.141	0.861	0.765
Without heterogeneous graph, using GIN and SAGPooling	0.135	0.890	0.801
Using dynamic heterogeneous graph with GIN and SAGPooling	0.123	0.908	0.821

**Table 7:** Specific details for three datasets used in this study.

	Proteins	Compounds	Binding/Interactions
Davis	442	68	30,056
KIBA	229	2111	118,254
Human	2001	2726	3369(+)/3359(−)

## Data Availability

All the relevant data are included within the paper.

## References

[1] Kairys V., Baranauskiene L., Kazlauskiene M., Matulis D., Kazlauskas E. (2019). Binding affinity in drug design: Experimental and computational techniques. Expert Opin. Drug Discov..

[2] Xue H., Li J., Xie H., Wang Y. (2018). Review of drug repositioning approaches and resources. Int. J. Biol. Sci..

[3] Berdigaliyev N., Aljofan M. (2020). An Overview of drug discovery and development. Future Med. Chem..

[4] Paul S.M., Mytelka D.S., Dunwiddie C.T., Persinger C.C., Munos B.H., Lindborg S.R., Schacht A.L. (2010). How to improve R&D productivity: The pharmaceutical industry’s grand challenge. Nat. Rev. Drug Discov..

[5] Liao J., Chen H., Wei L., Wei L. (2022). GSAML-DTA: An interpretable drug-target binding affinity prediction model based on graph neural networks with self-attention mechanism and mutual information. Comput. Biol. Med..

[6] Fan J., Fu A., Zhang L. (2019). Progress in molecular docking. Quant. Biol..

[7] Hollingsworth S.A., Dror R.O. (2018). Molecular dynamics simulation for all. Neuron.

[8] Mahesh B. (2020). Machine learning algorithms-a review. Int. J. Sci. Res..

[9] Jordan M.I., Mitchell T.M. (2015). Machine learning: Trends, perspectives, and prospects. Science.

[10] Alzubi J., Nayyar A., Kumar A. (2018). Machine learning from theory to algorithms: An overview. J. Physics Conf. Ser..

[11] Sahoo T.R., Patra S., Vipsita S. (2023). Decision tree classifier based on topological characteristics of subgraph for the mining of protein complexes from large scale PPI networks. Comput. Biol. Chem..

[12] Sindhwani M., Sachdeva S., Gupta A., Tanwar S., Alqahtani F., Tolba A., Raboaca M.S. (2023). A Novel context-aware Reliable routing protocol and svm implementation in vehicular area networks. Mathematics.

[13] Nascimento A.C.A., Prudêncio R.B.C., Costa I.G. (2016). A multiple kernel learning algorithm for drug-target interaction prediction. BMC Bioinform..

[14] He T., Heidemeyer M., Ban F., Cherkasov A., Ester M. (2017). SimBoost: A read-across approach for predicting drug–target binding affinities using gradient boosting machines. J. Cheminform..

[15] Chang C.H., Hung C.L., Tang C.Y. A review of deep learning in computer-aided drug design. Proceedings of the 2019 IEEE International Conference on Bioinformatics and Biomedicine (BIBM).

[16] Gawehn E., Hiss J.A., Schneider G. (2016). Deep learning in drug discovery. Mol. Inform..

[17] Lavecchia A. (2019). Deep learning in drug discovery: Opportunities, challenges and future prospects. Drug Discov. Today.

[18] Öztürk H., Özgür A., Ozkirimli E. (2018). DeepDTA: Deep drug–target binding affinity prediction. Bioinformatics.

[19] Ztürk H., Ozkirimli E., Özgür A. (2019). WideDTA: Prediction of drug-target binding affinity. arXiv.

[20] Chen L., Tan X., Wang D., Zhong F., Liu X., Yang T., Luo X., Chen K., Jiang H., Zheng M. (2020). TransformerCPI: Improving compound–protein interaction prediction by sequence-based deep learning with self-attention mechanism and label reversal experiments. Bioinformatics.

[21] Vaswani A. (2017). Attention is all you need. Adv. Neural Inf. Process. Syst..

[22] Zhang Z., Chen L., Zhong F., Wang D., Jiang J., Zhang S., Jiang H., Zheng M., Li X. (2022). Graph neural network approaches for drug-target interactions. Curr. Opin. Struct. Biol..

[23] Bongini P., Bianchini M., Scarselli F. (2021). Molecular generative graph neural networks for drug discovery. Neurocomputing.

[24] Xiong J., Xiong Z., Chen K., Jiang H., Zheng M. (2021). Graph neural networks for automated de novo drug design. Drug Discov. Today.

[25] Nguyen T., Le H., Quinn T.P., Nguyen T., Le T.D., Venkatesh S. (2020). GraphDTA: Predicting drug–target binding affinity with graph neural networks. Bioinformatics.

[26] Jiang M., Li Z., Zhang S., Wang S., Wang X., Yuan Q., Wei Z. (2020). Drug–target affinity prediction using graph neural network and contact maps. RSC Adv..

[27] Chu Z.Y., Huang F., Fu H.T., Quan Y., Zhou X.H., Liu S.C., Zhang W. (2022). Hierarchical graph representation learning for the prediction of drug-target binding affinity. Inf. Sci..

[28] Ma W., Zhang S., Li Z., Jiang M., Wang S., Guo N., Li Y., Bi X., Jiang H., Wei Z. (2023). Predicting drug-target affinity by learning protein knowledge from biological networks. IEEE J. Biomed. Health Inform..

[29] Yang Z., Zhong W., Zhao L., Chen C.Y.-C. (2022). MGraphDTA: Deep multiscale graph neural network for explainable drug–target binding affinity prediction. Chem. Sci..

[30] Nguyen T.M., Nguyen T., Le T.M., Tran T. (2021). Gefa: Early fusion approach in drug-target affinity prediction. IEEE/ACM Trans. Comput. Biol. Bioinform..

[31] Jiang M., Wang S., Zhang S., Zhou W., Zhang Y., Li Z. (2022). Sequence-based drug-target affinity prediction using weighted graph neural networks. BMC Genom..

[32] Zhang S., Tong H., Xu J., Maciejewski R. (2019). Graph convolutional networks: A comprehensive review. Comput. Soc. Networks.

[33] Wang S., Shan P., Zhao Y., Zuo L. (2021). GanDTI: A multi-task neural network for drug-target interaction prediction. Comput. Biol. Chem..

[34] Quan Z., Guo Y., Lin X., Wang Z.J., Zeng X. Graphcpi: Graph neural representation learning for compound-protein interaction. Proceedings of the 2019 IEEE International Conference on Bioinformatics and Biomedicine (BIBM).

[35] Zhong S., Guan X. (2023). Count-based morgan fingerprint: A more efficient and interpretable molecular representation in developing machine learning-based predictive regression models for water contaminants’ activities and properties. Environ. Sci. Technol..

[36] Davis M.I., Hunt J.P., Herrgard S., Ciceri P., Wodicka L.M., Pallares G., Hocker M., Treiber D.K., Zarrinkar P.P. (2011). Comprehensive analysis of kinase inhibitor selectivity. Nat. Biotechnol..

[37] Tang J., Szwajda A., Shakyawar S., Xu T., Hintsanen P., Wennerberg K., Aittokallio T. (2014). Making sense of large-scale kinase inhibitor bioactivity data sets: A comparative and integrative analysis. J. Chem. Inf. Model..

[38] Liu H., Sun J., Guan J., Zheng J., Zhou S. (2015). Improving compound–protein interaction prediction by building up highly credible negative samples. Bioinformatics.

[39] Bento A.P., Hersey A., Félix E., Landrum G., Gaulton A., Atkinson F., Bellis L.J., De Veij M., Leach A.R. (2020). An open source chemical structure curation pipeline using RDKit. J. Cheminform..

[40] Fey M., Lenssen J.E. (2019). Fast graph representation learning with PyTorch Geometric. arXiv.

[41] Paszke A., Gross S., Massa F., Lerer A., Bradbury J., Chanan G., Killeen T., Lin Z., Gimelshein N., Antiga L. (2019). Pytorch: An imperative style, high-performance deep learning library. NeurIPS.

[42] He H., Chen G., Chen C.Y.-C. (2023). NHGNN-DTA: A node-adaptive hybrid graph neural network for interpretable drug–target binding affinity prediction. Bioinformatics.

[43] Zhang Y., Hu Y., Han N., Yang A., Liu X., Cai H. (2023). A survey of drug-target interaction and affinity prediction methods via graph neural networks. Comput. Biol. Med..

[44] Rácz A., Bajusz D., Héberger K. (2018). Life beyond the Tanimoto coefficient: Similarity measures for interaction fingerprints. J. Cheminform..

[45] El-Din Rashed A.E., Amer H.M., El-Seddek M., Moustafa HE D. (2021). Sequence alignment using machine learning-based needleman–wunsch algorithm. IEEE Access.

[46] Velickovic P., Cucurull G., Casanova A., Romero A., Lio P., Bengio Y. (2017). Graph attention networks. STAT.

[47] Bouritsas G., Frasca F., Zafeiriou S., Bronstein M.M. (2022). Improving graph neural network expressivity via subgraph isomorphism counting. IEEE Trans. Pattern Anal. Mach. Intell..

[48] Lee J., Lee I., Kang J. (2019). Self-attention graph pooling. International conference on machine learning. Proc. Mach. Learn. Res..

